# Demographic and Urbanization Disparities of Liver Transplantation in Taiwan

**DOI:** 10.3390/ijerph15020177

**Published:** 2018-01-23

**Authors:** Pei-Hung Wen, Chin-Li Lu, Carol Strong, Yih-Jyh Lin, Yao-Li Chen, Chung-Yi Li, Chiang-Chin Tsai

**Affiliations:** 1 Department of Surgery, Cishan Hospital, Ministry of Health and Welfare, Kaohsiung 842, Taiwan; peihong@mail2000.com.tw; 2 Department of Public Health, College of Medicine, National Cheng Kung University, Tainan 701, Taiwan; chinli_lu@mail.ncku.edu.tw (C.-L.L.); carolcj@mail.ncku.edu.tw (C.S.); 3 Department of Surgery, College of Medicine, National Cheng Kung University, Tainan 701, Taiwan; lyj007@mail.ncku.edu.tw; 4 General Surgery Division, Surgery Department, Changhua Christian Hospital, Changhua City 500, Taiwan; 31560@cch.org.tw; 5 Transplant Medicine and Surgery Research Centre, Changhua Christian Hospital, Changhua City 500, Taiwan; 6 Department of Public Health, College of Public Health, China Medical University, Taichung 404, Taiwan; 7 Department of Surgery, Tainan Sin-Lau Hospital, Tainan 701, Taiwan; 8 Department of Health Care Administration, Chang Jung Christian University, Tainan 711, Taiwan

**Keywords:** liver transplantation, demographics, urbanization, descriptive epidemiology, prevalence rate

## Abstract

Limited access to or receipt of liver transplantation (LT) may jeopardize survival of patients with end-stage liver diseases. Taiwan launched its National Health Insurance (NHI) program in 1995, which essentially removes financial barriers to health care. This study aims to investigate where there are still demographic and urbanization disparities of LT after 15 years of NHI program implementation. Data analyzed in this study were retrieved from Taiwan’s NHI inpatient claims. A total of 3020 people aged ≥18 years received LT between 2000 and 2013. We calculated crude and adjusted prevalence rate of LT according to secular year, age, sex, and urbanization. The multiple Poisson regression model was further employed to assess the independent effects of demographics and urbanization on prevalence of LT. The biennial number of people receiving LT substantially increased from 56 in 2000–2001 to 880 in 2012–2013, representing a prevalence rate of 1.63 and 18.58 per 10^6^, respectively. Such increasing secular trend was independent of sex. The prevalence was consistently higher in men than in women. The prevalence also increased with age in people <65 years, but dropped sharply in the elderly (≥65 years) people. We noted a significant disparity of LT in areas with different levels of urbanization. Compared to urban areas, satellite (prevalence rate ratio (PRR), 0.63, 95% confidence interval (CI), 0.57–0.69) and rural (PRR, 0.76, 95% CI, 0.69–0.83) areas were both associated with a significantly lower prevalence of LT. There are still significant demographic and urbanization disparities in LT after 15 years of NHI program implementation. Given the predominance of living donor liver transplantation in Taiwan, further studies should be conducted to investigate factors associated with having a potential living donor for LT.

## 1. Introduction

Liver transplantation (LT) has rapidly advanced from an experimental therapy to a mainstream treatment option for a wide range of acute and chronic end-stage liver diseases (ESLD). LT has been considered as the most effective treatment modality for ESLD over the past five decades; and this procedure has evolved with refinement in surgical techniques and improvement in postoperative care [1]. While living donor liver transplantation (LDLT) quickly became the predominant form of LT in most Asian countries, it did not find such widespread acceptance in the Western societies. Such differences are primarily due to cultural, religious and political reasons. For example, the concept of brain death and organ donation was far less acceptable in many Asia countries compared to the West. Despite that, recent trends have shown that Asian societies have experienced a notable increase in deceased donor liver transplantation (DDLT) [1,2].

The prevalence of LT varied greatly across nations. In 2016, higher prevalence rates of LT were seen in numerous European countries such as Croatia (33.5 per 10^6^ people), Spain (25.2 per 10^6^ people), and Belgium (24.8 per 10^6^ people). Some countries outside of Europe also had higher prevalence rates of LT, including South Korea (27.8 per 10^6^ people) and United States of America (22.1 per 10^6^ people). On the other hand, United Kingdom (13.9 per 10^6^ people), Germany (11.1 per 10^6^ people), and Japan (3.6 per 10^6^ people) were developed countries with lower LT prevalence [3]. Factors such as prevalence of ESLD, criteria for LT, availability of donors, and waiting time for LT, all have contributed to the global variation in LT prevalence [4].

Survival has improved in recent decades among patients with LT. The overall 1-year survival rate currently approach 90%, whereas rates were <70% before 1980 [5]. Despite LT provides excellent long-term survival for individuals with ESLD, access to this life-saving procedure is usually not equal among those in need. Previous studies noted geographic variation in LT, in which rural residents had a greater impaired access to LT services [6,7]. Lower rates of access to LT also exist for racial minorities, women, and patients with lower socioeconomic status or inadequate insurance [8,9,10]. 

An Iranian study showed empirical evidence that a state coverage of the costs allowed people with lower educational attainment as well as those unemployed or unskilled workers to have a greater chance of receiving LT; and this policy also reduced socioeconomic inequality in utilization of LT [11]. Taiwan conducted the first successful DDLT in 1984, and later has become the first in Asia to frame legislation for organ donation [12]. The presence of universal coverage by the Taiwan National Health Insurance (NHI) program in 1995 enabled the establishment of clear guidelines for donor selection criteria, indications and timing for LT. There were 24 centers approved by the Ministry of Health and Welfare, which performed 3017 liver transplants in Taiwan between 2003 and 2012, with an overall 3-year survival rate of 82% [12].

Taiwan is a good setting for further examining the geographic differences in prevalence of LT, as the NHI program was intended to ensure the accessibility to health care at a reasonable cost [13]. The NHI has extended the health insurance coverage from 57% of the population (mostly the employed) to everyone; consequently, several segments of the population greatly benefited from the NHI program, including children, elderly people, and non-working adults [13]. Because lack of insurance has been associated with increased morbidity and mortality [14,15], implementation of the NHI program in Taiwan is expected to effectively remove the financial barriers to LT. After more than 1 decade of NHI program, this study aimed to explore the current demographic difference in LT prevalence, and to examine if there is still urban and rural disparity of LT prevalence in Taiwan. 

## 2. Materials and Methods

### 2.1. Source of Data

The study proposal was approved by the Institutional Review Board of National Cheng Kung University Hospital (A-EX-104-008). Data were retrieved from Taiwan’s NHI research database, a medical claim database that stores the medical records of beneficiaries that are uploaded by medical institutions to obtain reimbursement from NHI. Taiwan’s NHI program universally covers medical insurance for nearly all (>99%) Taiwanese citizens (prisoners and military personnel were exempted in our study period) [16]. The National Health Insurance Administration performs quarterly expert reviews on a random sample of medical claims to ensure their accuracy [17].

The present study was based on the inpatient claim data that include the records of all hospitalizations and provide various pieces of information, including encrypted personal identification number, date of birth, sex, dates of admission and discharge with one primary and up to four secondary diagnosis codes, operation codes if any, and type, location and accreditation of hospitals. The age and sex specific numbers of populations used to calculate the age-sex-specific prevalence rates of LT during the study period were obtained from Taiwan’s household registrations. 

### 2.2. Study Population

Based on the medical orders (liver organ receipt: 75022B 75021B, 75020B) and LT operation codes (ICD-9-CM, 50.5X, V59.6, V42.7, V42.9), we identified 3020 patients (2204 males and 816 females) hospitalized for LT at age of ≥18 years between 2000 and 2013. The overall and sex-specific biennial numbers of patients with LT from 2000–2001 to 2012–2013 are listed in Table 1.

### 2.3. Statistical Analysis

The biennial crude prevalence rate of LT was estimated by dividing the number of patients with LT by the total biennial number of Taiwanese population from 2000 to 2013. We also calculated, using the World Health Organization 2000 standard population, age- and sex-standardized biennial prevalence rates of LT [18]. The Poisson regression equation was employed to test whether a linear secular trend in LT prevalence exists over the study period. The age-standardized prevalence rates of LT over time were also calculated for both genders; and the age- and sex-specific prevalence rates were calculated from the weighted average of the annual prevalence rates of age and sex stratifications. We also calculated the age-sex-standardized prevalence rate of LT according to level of urbanization, namely, urban, satellite, and rural.

To account for the independent effects of age, sex, and calendar year as well as the potential effects of urbanization on LT prevalence, we conducted a multivariate Poisson regression analysis. The statistical analysis was performed using SAS version 9.4 (SAS Institute, Cary, NC, USA). A *p* < 0.05 was considered statistically significant. 

## 3. Results

**Figure 1:**
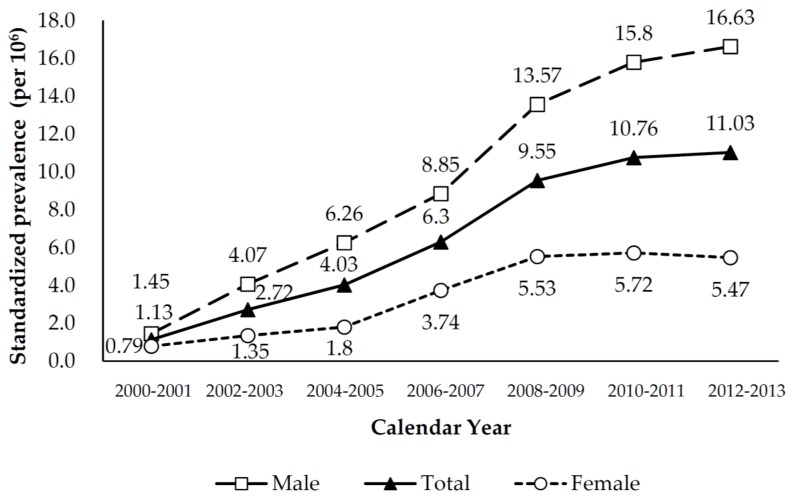
Overall (sex-age-standardized) and sex-specific (age-standardized) biennial prevalence of liver transplantation in Taiwan, 2000–2013.

**Figure 2:**
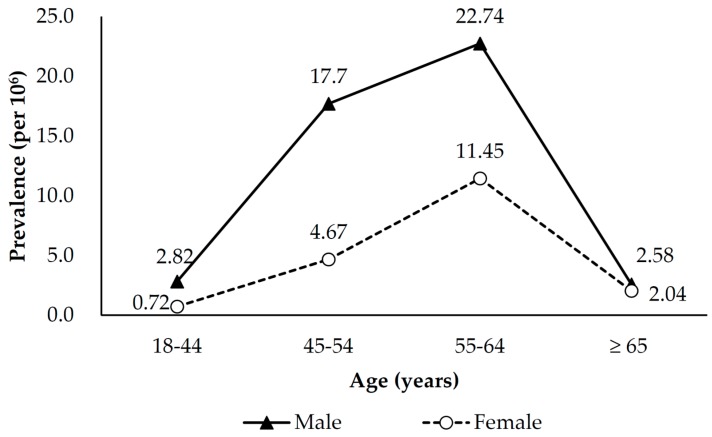
Sex-age-specific biennial prevalence of liver transplantation in Taiwan, 2000–2013.

Regardless of gender, the biennial number and prevalence rate of LT significantly increased between 2000 and 2013. The overall prevalence rate increased from 1.63 per 10^6^ in 2000–2001 to 18.58 per 10^6^ in 2013–2013, and the corresponding figures for men and women were 2.13 to 27.71 per 10^6^ and 1.13 to 9.51 per 10^6^. Men consistently had a higher prevalence rate of LT than women, with a male/female ratio ranging from 1.81 to 3.48. (Table 1). The age-sex-standardized prevalence rate also shows an uprising trend. Such increasing trend was noted in men, but not in women whose prevalence appeared to have reached a plateau in 2008–2009 (Figure 1). The prevalence rate of LT increased with age before 64 years; and it reached the highest prevalence in people aged 55–64 years irrespective of gender. The prevalence rate dropped rapidly after 64 years in both men and women (Figure 2).

**Figure 3:**
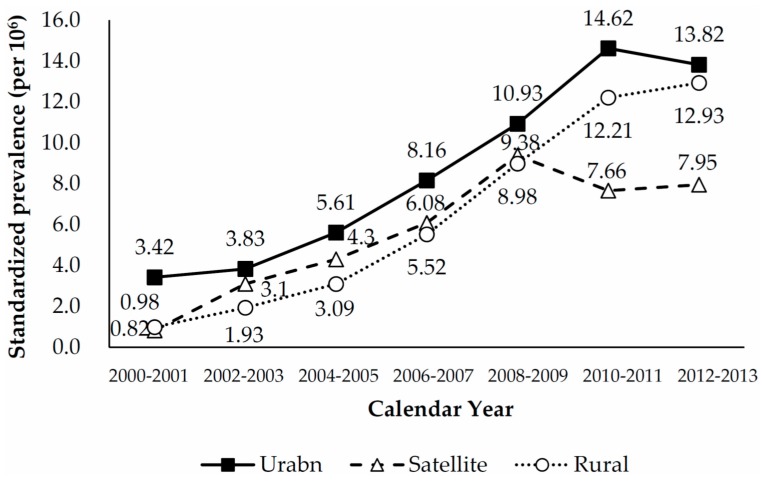
Sex-age-standardized biennial prevalence of liver transplantation in Taiwan, 2000–2013, according to level of urbanization.

**Figure 4:**
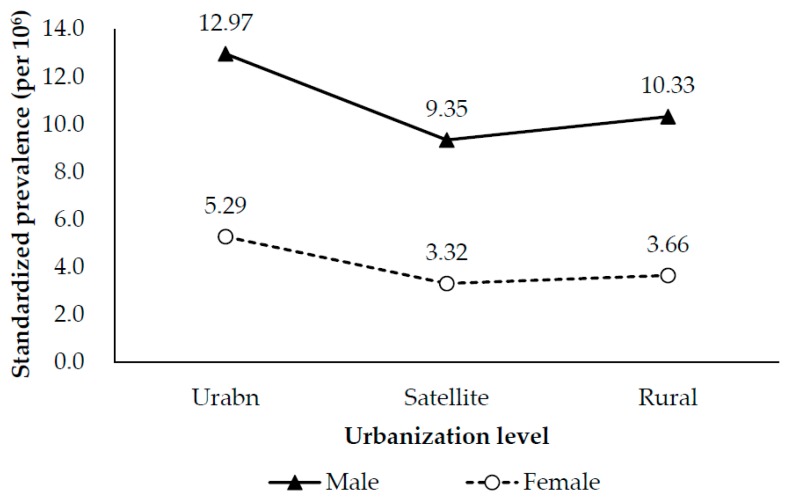
Age-standardized biennial prevalence rates of liver transplantation for men and women living with different levels of urbanization.

Table 2 shows crude and age-sex-standardized biennial prevalence rates of LT, according to level of urbanization. On additive scale, both urban and rural areas were associated with a greater increase in crude and standardized prevalence rates than were satellite areas. On multiplicative scale, however, the changes in crude and standardized prevalence rates were much higher in rural areas than in urban and satellite areas. The standardized prevalence rate is consistently higher in urban areas than in satellite or rural areas over the study period. Additionally, the prevalence rate was higher in satellite areas than in rural areas between 2000 and 2009. However, the prevalence rate in satellite areas slightly dropped after 2009, and became lower than that of rural areas (Figure 3). For both men and women, the highest and lowest age-standardized prevalence rates were noted in urban and satellite areas, respectively (Figure 4).

Table 3 shows the results from multivariate Poisson regression analysis. Compared to 2000–2001, the prevalence rate ratio (PRR) of LT increased over time, with the highest PRR noted in 2012–2013 (9.68, 95% Confidence Interval (CI), 7.39–12.69). Compared to people <45 years, all older age groups were associated with a significantly elevated IRR at 5.93 (45–54 years), 8.42 (55–64 years), and 1.23 (≥65 years), respectively. Additionally, men are more likely to receive LT than women (PRR = 2.74, 95% CI, 2.53–2.97). Moreover, the IRR of LT also varied with level of urbanization. Compared to urban areas, satellite (PRR, 0.63, 95% CI, 0.57–0.69) and rural (PRR, 0.76, 95% CI, 0.69–0.83) areas were both associated with a significantly reduced PRR.

## 4. Discussion

This nationwide population-based study demonstrated a substantial increase in number and prevalence of LT in 2000–2013. However, apart from obvious age and gender disparities of LT prevalence, we also noted a significant and positive relationship between level of urbanization and prevalence of LT during the era of universal health insurance coverage in Taiwan.

Our study results were in line with some prior study findings suggesting lower prevalence of LT in rural areas. The reasons behind this phenomenon could be complex and multifocal. The study by Arnon et al. noted that compared with urban residents, waiting list registration rates for rural/small town residents were significantly lower for LT; and wait-list mortality was also higher for the public insurance group than for the private insurance group [19]. The study by Axelrod et al. noted that patients living in rural areas had a lower rate of wait-listing and transplant of various solid organs including liver [20]. Besides, patients who live in rural regions and small towns usually face multiple barriers to health care access, including the need to travel long distances, the lack of locally available specialty services, and difficulty in receiving follow-up care [21]. The study by Doyle et al. reviewed 491 consecutive patients listed for LT and compared between those who had a potential living donor volunteer for assessment and those who did not. It found that patients with a living donor were more likely to have Child-Pugh C disease, and less likely to be older, single, divorced, immigrants, or from the lowest income quintile [22].

Like many parts of East Asia, the concept of brain death and organ donation was far less acceptable in Taiwan compared to the West. The prevailing cultural values attached enormous importance to families and ancestors. The attitude that cadaveric donation would desecrate the deceased person’s spirit, combined with dismal outcomes in certain countries, resulted in considerable reluctance to get involved with organ donation [12]. Because of this, there was widespread acceptability of the idea of LDLT in East Asia, including Taiwan where more than 80% of LT are LDLT [23]. On the contrary, the proportion of LDLT was much lower in the Western societies. For example, the prevalence of LDLT and DDLT was 0.95 and 17 per 10^6^ people in the United States in 2010. The corresponding figures for Spain were 0.7 and 24.5 per 10^6^ people [24].

Although LDLT may shorten the waiting time and decrease the risk of dropping off the waiting list because of death or disease progression, there have been strict regulations on performing LDLT in Taiwan. According to Taiwan Human Organ Transplant Act, adults or married minors aged over 18 years are allowed to donate part of their liver organ to relatives no more than fifth degree of kinship. In addition, unmarried minors aged over 18 years may donate part of their liver organ to blood relatives of no more than fifth degree of kinship with the written consent of their statutory representative [25,26]. Previous Taiwanese studies showed a clustering of viral hepatitis in families of patients with chronic liver diseases, likely due to infection and common genetic origins [27,28,29]. Such family clustering in liver disease might have further comprised the availability of living donors for ESLD patients in Taiwan. This could also contribute, at least to some extent, to the urban-rural difference in LT prevalence noted in this study, because chronic ESLD due to viral hepatitis was the commonest indication for adult LDLT in Taiwan [12]. Moreover, the prevalence of and risk factors for viral hepatitis were higher in rural than in urban areas [30]. Patients from rural areas might thus face considerable barriers to receive LDLT.

Like many other previous studies [8,9,10], our data also suggested an obvious demographic variation in prevalence of LT. A higher LT prevalence noted in men could be largely due to a higher prevalence of ESLD in male population. A recent Taiwanese study found that men aged 30–69 years had significantly higher risks of chronic liver disease, cirrhosis, and hepatocellular carcinoma than their female counterparts, with a covariate adjusted rate ratio of 4.33, 4.23, and 3.60, respectively [31]. The sex disparity of prevalence of chronic liver disease, cirrhosis and hepatocellular carcinoma is mainly due to gender behaviors or environmental impact, in which the prevalence of alcohol consumption, smoking, and occupational exposure to certain solvent with liver toxicity was usually higher in men than in women. In addition, the sex disparity of Hepatitis B virus (HBV)-related liver diseases including hepatocellular carcinoma has been noticed for a long time, which could be attributed to sex hormone effects [32]. The prevalence of LT increased with age among young and middle-aged group, but sharply decreased in elderly population. It is not uncommon that for the elderly patients, conservative treatment may sometimes be preferable, particularly for frail individuals who are more vulnerable to treatment-related complications [33,34]. Additionally, because the elderly people are often underrepresented in clinical trials, treatment guidelines that are based on these trials are unable to provide recommendations specifically for the very elderly. Given a rapid increase in size of elderly population in many industrialized societies, careful evaluation of LT need in elderly population is warranted. 

This is the first population-based study conducted in Taiwan to describe the demographic and urbanization variations in LT. Data analyzed in this study were retrieved from the NHI claims data, which have considered representative [35]. Although LT was based on the procedure codes, the likelihood of information bias is small as LT is a procedure requiring detailed documentation for insurance reimbursement. 

## 5. Conclusions

In conclusion, despite an increase in numbers and rates of LT in Taiwan in the era of universal coverage of health insurance, there are still significant urban-rural differences in LT prevalence. A lower prevalence of LT in rural areas may be indicative of non-financial barriers to LT in these areas. Apart from the factors attributable to the limited access to DDLT among those socioeconomically disadvantageous people, limited availability of healthy living donors in ESLD patients in rural areas could also contribute to the observed urban-rural difference in LT. Health policy makers should take into account our study findings in reallocating the LT resources to further reduce the urban-rural disparity of LT in Taiwan.

## Figures and Tables

**Table 1:** Overall and sex-specific biennial numbers and rates of liver transplantation in Taiwan, 2000–2013.

Calendar Year	Total		Men		Women		M/F Ratio ^b^
	No. of Cases	Prevalence ^a^	No. of Cases	Prevalence ^a^	No. of Cases	Prevalence ^a^	
2000–2001	56	1.63	37	2.13	19	1.13	1.88
2002–2003	139	3.95	105	5.89	34	1.95	3.02
2004–2005	213	5.92	166	9.15	47	2.63	3.48
2006–2007	353	9.57	250	13.51	103	5.60	2.41
2008–2009	566	14.99	400	21.22	166	8.78	2.42
2010–2011	813	17.50	592	25.49	221	9.51	2.68
2012–2013	880	18.58	654	27.71	226	9.51	2.91
Total	3020		2204		816		
*p* for trend		<0.001		<0.001		<0.001	

^a^ per 10^6^. ^b^ M/F ratio: prevalence in men/prevalence in women.

**Table 2:** Crude and age-sex-standardized biennial prevalence of liver transplantation in Taiwan, 2000–2013.

Level of Urbanization	Calendar Year							Absolute Change (10^6^)	Relative Change (%)
	2000–2001	2002–2003	2004–2005	2006–2007	2008–2009	2010–2011	2012–2013		
Urban									
Crude	3.56	5.78	8.53	12.83	17.77	24.23	24.03	20.5	6.8
Standardized ^a^	2.42	3.83	5.61	8.16	10.93	14.61	13.82	11.4	5.7
Satellite									
Crude	1.25	4.46	6.27	9.20	14.84	12.40	13.24	12	10.6
Standardized ^a^	0.82	3.10	4.30	6.08	8.98	7.66	7.95	7.1	9.7
Rural									
Crude	0.98	2.76	4.48	8.31	13.82	19.71	21.62	20.6	22.1
Standardized ^a^	0.98	1.93	3.09	5.52	9.38	12.21	12.93	12	13.2

^a^ Age and sex adjusted prevalence rate, per 10^6^.

**Table 3:** Associations of calendar year, demographics, and urbanization with rate of liver transplantation.

	People with Liver Transplantation			Adjusted Prevalence Rate Ratio		
	No.	Prevalence ^a^	Crude Prevalence Rate Ratio	Estimates	95% CI	*p*-Value
Calendar year						
2000–2001	56	1.63	1	1		
2002–2003	139	3.95	2.42	2.35	1.72–3.21	<0.0001
2004–2005	213	5.92	3.63	3.41	2.54–4.58	<0.0001
2006–2007	353	9.57	5.87	5.33	4.02–7.06	<0.0001
2008–2009	566	14.99	9.20	8.06	6.13–10.61	<0.0001
2010–2011	813	17.50	10.74	9.34	7.13–12.25	<0.0001
2012–2013	880	18.58	11.40	9.68	7.39–12.69	<0.0001
Age (years)						
<45	546	3.58	1	1		
45–54	1174	22.42	6.26	5.93	5.36–6.57	<0.0001
55–64	1136	34	9.50	8.42	7.60–9.33	<0.0001
≥65	164	4.61	1.29	1.23	1.03–1.46	0.02
Sex						
Women	816	5.98	1	1		
Men	2204	16.02	2.68	2.74	2.53–2.97	<0.0001
Level of urbanization						
Urban	1319	14.08	1.32	1		
Satellite	911	9.64	0.90	0.63	0.57–0.69	<0.0001
Rural	790	10.70	1	0.76	0.69–0.83	<0.0001

CI, Confidence Interval. ^a^ per 10^6^.
